# Digital decision aid for prenatal counseling in imminent extreme premature labor: development and pilot testing

**DOI:** 10.1186/s12911-021-01735-z

**Published:** 2022-01-06

**Authors:** Josephus F. M. van den Heuvel, Marije Hogeveen, Margo Lutke Holzik, Arno F. J. van Heijst, Mireille N. Bekker, Rosa Geurtzen

**Affiliations:** 1 Department of Obstetrics, Wilhelmina’s Children Hospital, University Medical Center Utrecht, Utrecht, The Netherlands; 2 Department of Neonatology, Amalia Children’s Hospital, Radboud University Medical Center, Internal Code 804, PO Box 9101, 6500HB Nijmegen, The Netherlands; 3 Department of Obstetrics, Leiden University Medical Center, Leiden, The Netherlands

**Keywords:** Digital health, Neonatology, Obstetrics, Patient decision aid, IPDAS, Shared decision making, Prematurity

## Abstract

**Background:**

In case of extreme premature delivery at 24 weeks of gestation, both early intensive care and palliative comfort care for the neonate are considered treatment options. Prenatal counseling, preferably using shared decision making, is needed to agree on the treatment option in case labor progresses. This article described the development of a digital decision aid (DA) to support pregnant women, partners and clinicians in prenatal counseling for imminent extreme premature labor.

**Methods:**

This DA is developed following the International Patient Decision Aid Standards. The Dutch treatment guideline and the Dutch recommendations for prenatal counseling in extreme prematurity were used as basis. Development of the first prototype was done by expert clinicians and patients, further improvements were done after alpha testing with involved clinicians, patients and other experts (n = 12), and beta testing with non-involved clinicians and patients (n = 15).

**Results:**

The final version includes information, probabilities and figures depending on users’ preferences. Furthermore, it elicits patient values and provides guidance to aid parents and professionals in making a decision for either early intensive care or palliative comfort care in threatening extreme premature delivery.

**Conclusion:**

A decision aid was developed to support prenatal counseling regarding the decision on early intensive care versus palliative comfort care in case of extreme premature delivery at 24 weeks gestation. It was well accepted by parents and healthcare professionals. Our multimedia, digital DA is openly available online to support prenatal counseling and personalized, shared decision-making in imminent extreme premature labor.

**Supplementary Information:**

The online version contains supplementary material available at 10.1186/s12911-021-01735-z.

## Background

Pregnant women and their partners presenting with imminent extreme premature labor are facing treatment options regarding the provided care in case their infant is born alive: palliative comfort care or early intensive care. A so-called “gray zone of parental discretion” exists, in which parents and physicians make treatment decisions together [1–5]. The exact definition of this zone, in terms of i.e. gestational age, changed over time. Furthermore, this gray zone can differ across countries, health systems and cultures [4, 6–8]. Despite different definitions of this gray zone of parental discretion, parental counseling is needed to agree on the preferred treatment option, ideally based on shared decision making between the pregnant woman, her partner (*further referred to as parents*), obstetrician and neonatologist [9–12]. Given the risks of morbidity and mortality in extremely premature infants, both palliative comfort care and early intensive care are considered treatment options [2, 4, 9–12]. This emphasizes the importance of involving parents in decision-making. Palliative comfort care can be described as providing warmth and comfort to the newborn but no medical assistance and acceptance of the death of the infant. Early intensive care can be described as the resuscitation of the premature infant and the initiation of neonatal intensive care.

Decision aids (DAs) are used to assist in shared decision-making, as they provide objective information and visual material in a comprehensive way. DAs have been proven to be effective to enhance communication between patient and physician by eliciting situation-specific values and preferences. Moreover, they improve patient knowledge and satisfaction in the shared decision-making process for both patients and care providers [13, 14].

Internationally, some tools have been developed to help parents in the situation of imminent extremely premature labor. An overview of these tools is provided in the supplementary material (Additional file 1), and includes visual aids, card sets, videos and a (tablet) application, all from Northern America. Five of seven studies iregarding these tools are (partly) based on one developmental study from 2012 by Guillen et al. [15]. In six of seven described tools, parents of children born extreme prematurely were involved in the design process. All tools were well-accepted amongst their users. Most published DA tools were developed to aid in the hand-over of medical information rather than helping parents’ to explore their values and preferences [4]

In the Netherlands, counseling at the limits of viability is preferably performed in tertiary perinatal care centers, by an obstetrician and neonatologist together. The most recent Dutch treatment guideline, in use since 2010, stated that “informed consent of parents is prerequisite in the decision whether or not to initiate care at 24 weeks gestational age”[16]. In addition, nationwide recommendations to support parental counseling were published in 2019, following a Delphi consensus method (a structured and interactive method that relies on a panel of experts to build consensus in several rounds) [12]. The model of shared decision making (SDM) was recommended because of the preference-sensitive nature of the treatment decisions at the lower limits of viability, similar to international recommendations [2, 4, 9, 11, 12]. Furthermore, both in the Netherlands and internationally, there was a growing demand and recommendation for supportive material in the counseling, in addition to the verbal conversation [9, 17–19].

We developed a DA to (a) fulfill the need for visual supportive material, (b) take into account the Dutch situation, guideline, and language, and (c) to better involve parents in decision-making, using value elicitation. Aim of this research was to describe the development (process, content and pilot-testing) of this DA to support prenatal counseling in imminent extreme premature labor.

## Methods

### Setting

In the Netherlands, nine perinatal care centers with NICU (neonatal intensive care unit) facility exist. In Dutch practice since 2010, the defined gray zone of parental discretion lies between gestational age (GA) of 24^0/7^ and 24^6/7^, but counseling can be offered from 23^0/7^ onwards [12, 16]. Each year approximately 170.000 children are born, of which approximately 150 between 24^0/7^ and 24^6/7^ weeks GA [20]. Since the number of patients presenting with imminent extreme premature labor outreaches the actual number of extreme premature birth, the amount of antenatal counseling is multiple times more frequent than 150/year.

### Development

**Fig. 1:**
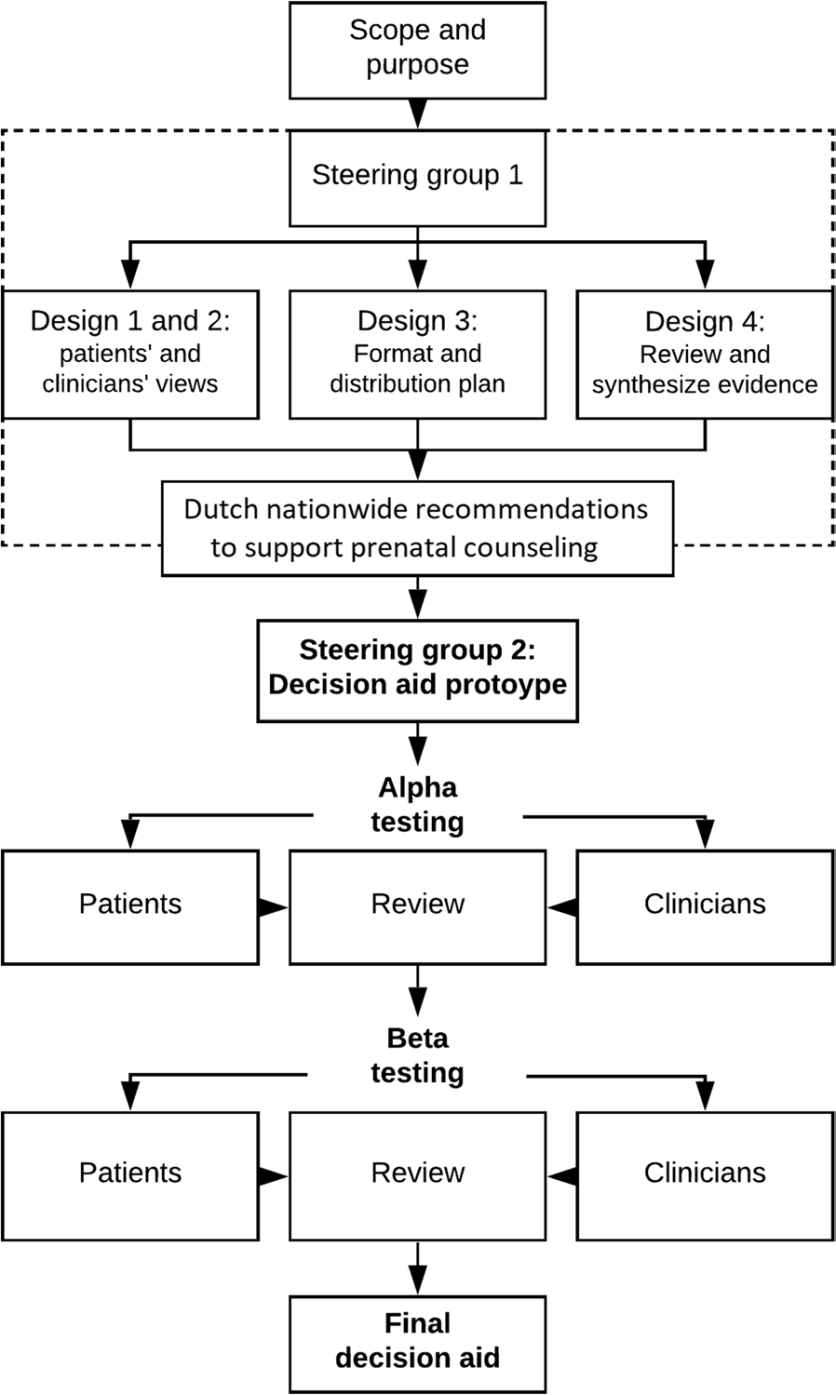
Developmental process of the decision aid. Based on Coulter et al. [21] with adjustments specific for our process. The dotted box represents the earlier work for the development of the Dutch recommendations for prenatal counseling in extreme prematurity [12]

The digital DA was developed using a model development process that includes all original elements of the systematic process established by the International Patient Decision Aid Standards (IPDAS)[21].This process is summarized in Fig. 1 and each stage is described below. Several parts within the IPDAS process were already covered in the development of the Dutch counseling recommendations and are indicated by the dotted box in Fig. 1[12].

#### Scope and purpose

The purpose of this digital DA was to support both expectant parents and physicians in prenatal counseling regarding treatment options for imminent extremely premature birth. Next to obstetricians and neonatologist, the target audience comprises expectant parents of the general Dutch population, who will receive prenatal counseling before 25^0/7^ weeks GA. Considering potential birth at 24^0/7^- 24^6/7^ weeks, counseling and thus use of the DA may take place from 23^0/7^ weeks onwards [12]. The information in de DA was limited to imminent extreme prematurity, of a singleton, following spontaneous preterm labor, since that was the scope of the Dutch treatment guideline [16]. So, this DA was not developed for use in multiple pregnancy, prenatally diagnosed congenital anomalies and/or intended iatrogenic preterm delivery (e.g. in case of [severe] fetal growth restriction or maternal morbidly such as [severe] preeclampsia).

#### Steering group 1 (previous work)

For the development of the Dutch counseling recommendations, an expert panel was formed for a Delphi consensus study – which can be regarded as steering group 1. It consisted of 6 (pairs of) parents, 7 obstetricians and 8 neonatologists (total n = 21)[12]. Those parents all experienced premature birth at 24^+0/7^ – 24^+6/7^ weeks GA between 2010 and 2013 and received counseling in 5 different hospitals. Regarding the 6 experienced (pairs of) parents, palliative comfort care was chosen once and early intensive care was chosen 5 times. From their 9 infants, 3 survived. All 9 tertiary perinatal care centers in the Netherlands were represented by at least an obstetrician or neonatologist or both in the steering group. Both the Delphi consensus study, and earlier work among Dutch parents and physicians [17, 18, 22, 23], are comparable to the first stages (design 1 to 4) in the IPDAS development process and were therefore not repeated. This is indicated with a dotted box in Fig. 1 and summarized below.

#### Patients’ and clinicians’ views (design 1 + 2)

Patients’ views on prenatal counseling were assessed before in a survey (n = 61) and interviews (n = 13) [18, 23], just as clinicians’ views in a survey (n = 122) and (focus group) interviews (n = 35) [17, 22]. Both patients and clinicians wished to use supportive material in the counseling, in addition to the verbal conversation. However, they expressed a lack of availability of suitable material. Parents asked for visualization of complex information with optional, expandable boxes for further details or numbers [18]. Both treatment options (early intensive care and palliative comfort care) should be described in understandable, neutral language and consensus was reached on the most important information needed to make this decision [12].

#### Format and distribution (design 3)

Next to their general views and preferences regarding prenatal counseling, both clinicians and parents were specifically assessed on their preferences regarding a DA in the interviews as referred to in design 1 and 2[17, 18]. As patients with imminent preterm labor present to the hospital unannounced, the DA must be available instantly to support counseling. It was decided that the decision support must be available online without need for login and free of charge.

#### Review and synthesize evidence (design 4)

In a Delphi consensus method, evidence from international literature was combined with Dutch results (patients’ and clinicians’ views from Design 1–2) to develop cultural sensitive, nationwide recommendations for prenatal counseling in extreme prematurity [12, 17, 18, 22, 23].This was used, together with the Dutch guideline for perinatal management of extremely preterm delivery, to compose the main part of the DA [16].

#### Steering group 2: decision aid prototype

For the development of the DA, a new steering group was created. This group consisted of 2 obstetric professionals (JvdH, MB) and 2 neonatal professionals (RG, MH), representing two tertiary perinatal centers, and one professional in development of DAs. Steering group 2 drafted the first version of the DA (see Results). This first prototype was checked and refined using the IPDAS criteria before pilot testing.

### Pilot testing

#### Alpha testing

To check comprehensibility and usability of the first draft, several rounds of alpha testing were performed, in an iterative process during development with members of our Steering groups. Involved in this stage were six parents of children born extremely premature (born between 24^0/7^ weeks and 24^6/7^ weeks, previously involved in stage Design 1). Furthermore, six other experts were involved: two obstetricians (representing two different perinatal centers), one neonatologist, an expert in quality of care improvement, a professional in DA development and one Dutch language expert of the Easy Reading Platform (in Dutch: Stichting Makkelijk Lezen). The latter was involved to assure readability of the (Dutch) text following their “Quality Seal for comprehendible texts”. All participants in alpha testing (total n = 12) reviewed the content of the DA several times, leading to multiple rounds of revision. Steering group 2 reviewed all comments and finalized the process of alpha testing. The first prototype was subsequently build in the online module, including graphics, for beta testing.

#### Beta testing

Feasibility of the DA was checked during one round of beta testing with clinicians and pregnant women, in a “real-world” setting, outside of the developmental process. All participants (total n = 15) were not previously involved in the processes of the DA development. Patient participants comprised two groups. One group involved pregnant women between 24^0/7^ and 24^6/7^ weeks GA, presenting to our outpatient clinic for a regularly scheduled antenatal visit, without (history of) signs of prematurely delivery (n = 4). The second group involved pregnant women, who were admitted for imminent preterm labor < 24^6/7^ weeks GA and received antenatal counseling (n = 3). Clinician participants were obstetricians and neonatologists recruited amongst two Dutch university hospitals with NICU facility (n = 8, 4 obstetricians and 4 neonatologists). All reviewed the online module of the digital DA and subsequently filled out a questionnaire. This questionnaire included 7 propositions to be rated on a 5 point Likert scale (very much disagree – very much agree) and two open questions, asking for three positive points of the concept DA and three improvement points. Answers to the multiple choice and open questions were reviewed by Steering group 2 and discussed in order for improvement of the final version of the DA.

## Results

### Information in the decision aid

Recommendations regarding important content for decision-making [12] were incorporated in this first prototype, being: explanation of treatment options, information on survival, risk of permanent consequences, impossibility to predict an individual course, possibility for multiple future decision moments, and parental values and standards [12]. Different steps of perinatal counseling functioned as an outline for the construction of the DA, and this is also the order in which the information is presented to the users. The first 6 steps were based on what was considered most important for clinical decision-making by patients and clinicians, step 7 to 9 were based on IPDAS criteria [12, 21, 24], and outlined in Table 1.

**Table 1:** Outline of the different steps of the DA for perinatal counseling

Step	Information in the Decision Aid
1	General information on imminent extreme premature labor
2	Explanation of the two options: early intensive care or palliative comfort of the neonate
3	Consequences of early intensive care after birth (such as: NICU admission, ventilation but also social consequences)
4	Consequences of comfort care (such as: no need for invasive procedures, expected death within hours after birth)
5	Information on mortality rates
6	Risks and long-term results of extreme premature infants on neurodevelopmental and physical level, as well as visual and hearing problems

	Decision making support with the Decision Aid
7	Comparison page
8	Important points
9	My choice

**Fig. 2:**
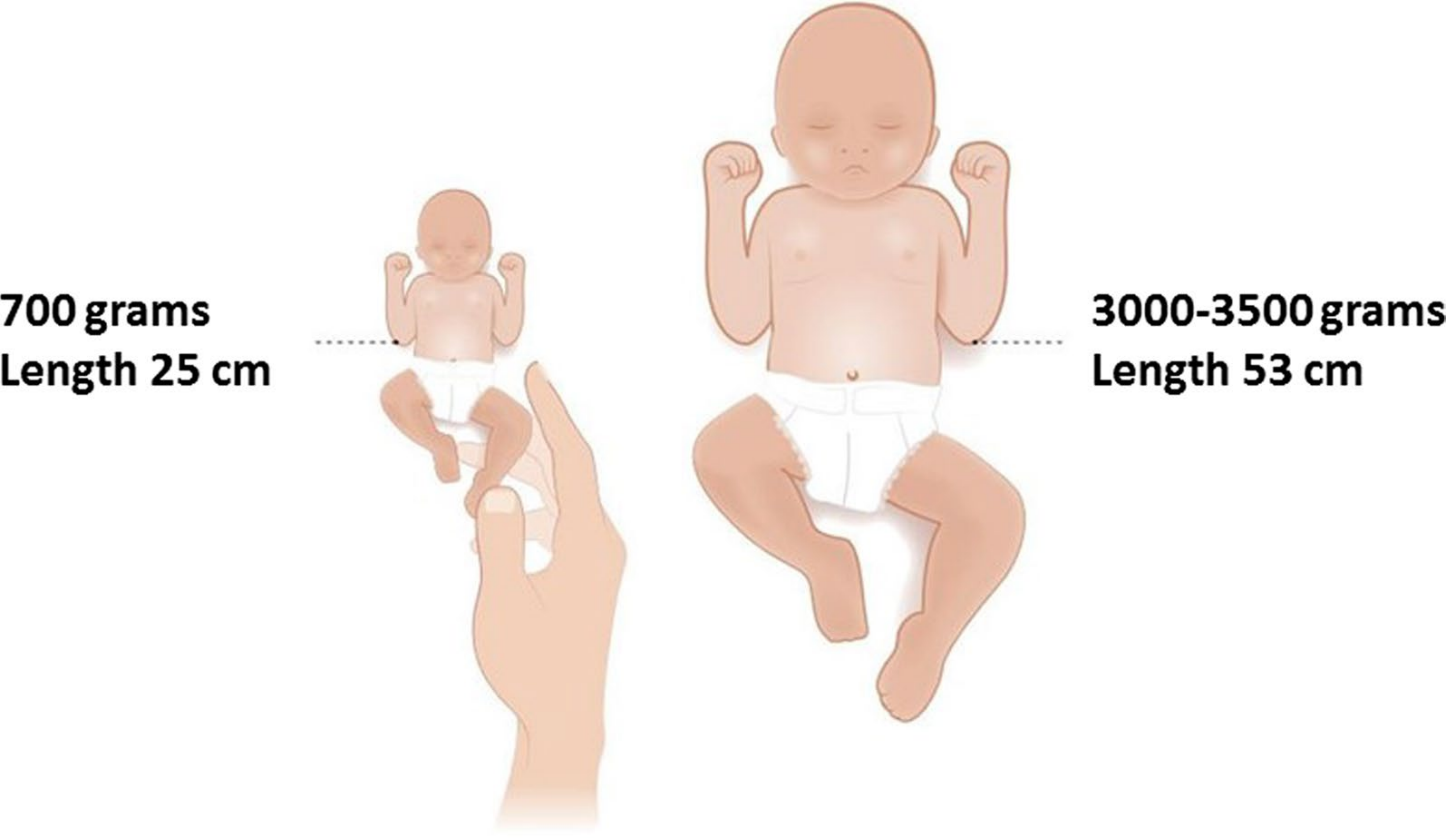
Illustration of two neonates, used in Step 1 of the DA

**Fig. 3:**
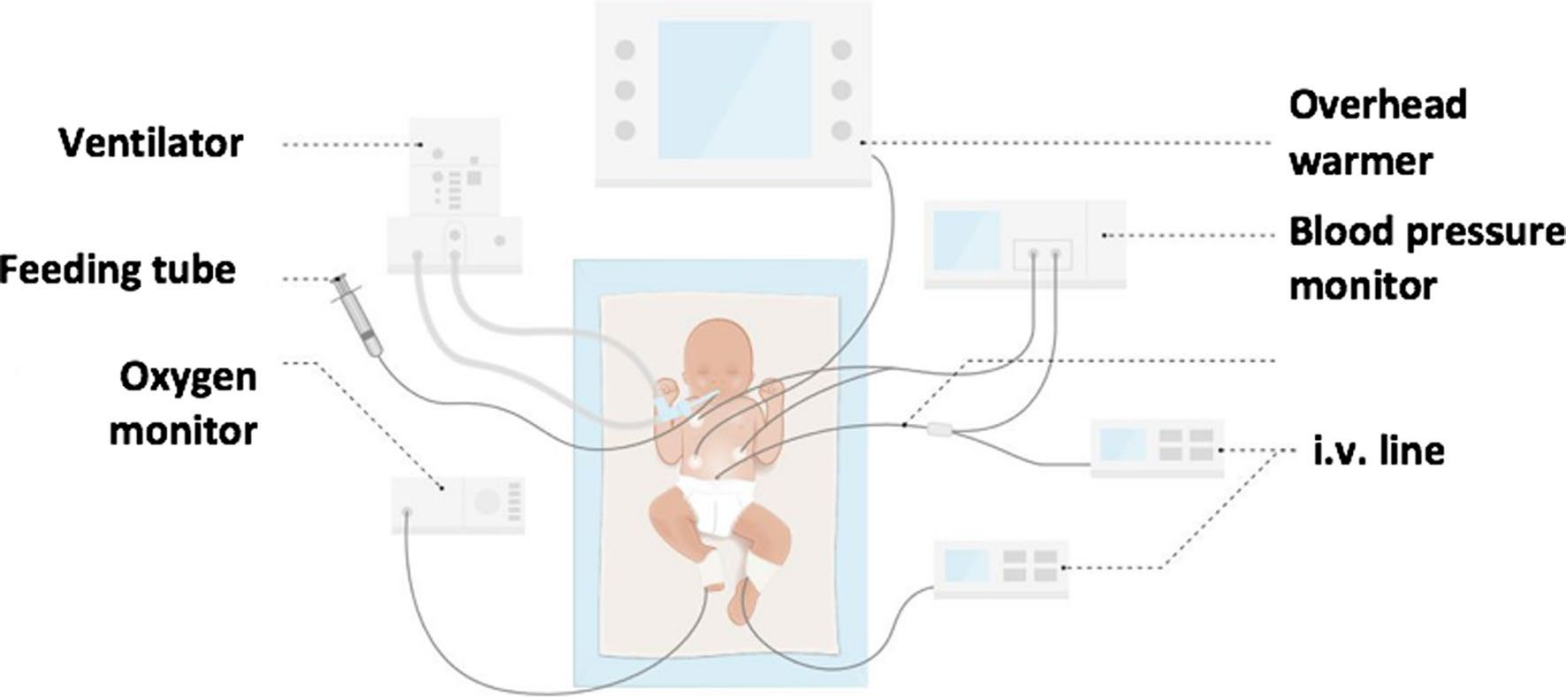
Illustration of Step 3 of the DA, a premature neonate in an incubator with explanations

Mortality and survival rates of children born before 24^6/7^ weeks GA were based on the national perinatal registry (Perined) between 2011–2017 [20], since national data were preferred [12, 23]. Long-term morbidity rates were derived from the international morbidity data overview as summarized in the Dutch guideline and two Dutch publications on this matter [16, 25, 26] – not entirely Dutch data could be used here because of the lack of a large Dutch cohort. Information on mortality and survival and adverse long-term outcome was illustrated with pictographs expressed as number per 100 neonates [11]. Graphic illustrations were included in several steps of the DA, including an illustration of two neonates in order to compare size and weight after extreme preterm and term delivery (Fig. 2 and Supplementary material—Additional file 2), an extreme premature infant in an incubator with explanation of all life-supporting devices (Fig. 3), and illustrations to support the content on possible disabilities. Figures, pictures and illustrations were partly inspired by [15, 27], and partly shared by [27] previous international publications. A picture of an extreme premature infant in an incubator (active care) and one covered in blankets held by the parent (comfort care) was used following informed consent of parents involved.

### Decision making support with the decision aid

Three additional steps were developed to aid in decision making (Table 1). In step 7, the “Comparison” page includes a table to compare active care and comfort care with regards to short-term and long-term consequences, and their disadvantages and complications. The addition of stage 8 is an “Important points” page. This page contains 5 yes / no questions with immediate visible feedback after users answer one of the questions. With these questions, users can test their understanding of key issues presented in the DA. Stage 9 is the “My choice” page, which includes a values clarification exercise with 7 propositions to weigh the two different treatment options in line with the users’ values (Supplementary material, Additional file 2). Answering the questions of knowledge or values however is not obligatory and the DA does not give a ‘preliminary’ decision or advice based on these additional features of the DA. The concluding page contains a statement to encourage patients to discuss questions of the content and personal values with their clinicians.

### Alpha testing

Alpha testing comprised multiple rounds with 12 participants for revision of the text of the main body and the illustrations. The revisions concerned mostly clarity of wording, phrasing and shortening without compromising the actual content. The level of readability was comparable to eight grade level in English language.

Two visual aspects of the DA raised discussion in the rounds of alpha testing. Regarding the report of numbers of mortality and morbidity, two options were explored. The first option was to show all numbers, including the pictographs, directly in the main body of the DA. The second option was to explain the possibility to review numbers and pictographs, followed by a mouse-click in an expanding drop-down box. There was consensus on the second option after exploration of both parents’ and clinicians’ views. This is consistent with previous literature describing that many, but not all, parents would like to review statistics [12, 18, 28]. Second, the level of detail that should be presented to all users was discussed. Since the various needs for detail and technical information among parents [5, 18], we choose to also apply optional expandable boxes, for example to several graphics, images of procedures and images of neonates with both intensive as well as palliative care.

### Beta testing

After alpha testing, the first prototype of the DA as website was tested (n = 15). All 15 participants reviewed the digital DA and answered the usability questionnaire. Results are shown in Table 2 and showed consistent positive impressions from both clinicians and patients on themes as information provision and ease of use. Open comment fields of positive points highlighted the quality and value of the illustrations and pictographs (4 clinicians and 3 patients), the “My choice” page for value clarification (3 clinicians and 2 patients) and the readability of the text (4 patients). Besides some textual improvements, suggestions for improvement from clinicians’ point of view were: more information needed on obstetrical care (treatment with tocolytics, fetal lung maturation and mode of delivery), and the assurance of updating (national) mortality and morbidity statistics with future analysis of extreme prematurity outcomes. Although one participant mentioned that the “Comparison” page was unnecessary as it contained repeated information, the Steering group decided to leave this page in.

**Table 2:** Results of usability questionnaire amongst clinicians and patients

Statement	Clinicians N = 8 Median	Patients N = 7 Median
The length of the DA was appropriate	4	4
The information presented in the DA was formulated too negatively	2	3
The information presented in the DA was presented too positively	1.5	2
The DA provided useful information on premature birth	4	4
The DA provided useful insight on how premature babies grow up in the future	4	4
I would like to use the DA if I was in the situation facing extreme premature birth	4	4
The DA was easy to use	4	4.5
The information of the DA was provided in a synchronized order	4	4
I think most people can easily use the DA	4	4

1 = very much disagree, 2 = disagree, 3 = no opinion, 4 = agree, 5 = very much agree, *DA* = *decision aid*

### Finalizing the DA

Results from field testing including all open comments were reviewed by the research group and several textual changes were made, which led to the final version of the DA. All IPDAS criteria were fulfilled (Additional file 3)[24].Using a website as platform, patients and clinicians from all nine Dutch tertiary perinatal centers can make use of the DA at any time of day. The website is built for internet browsers, as well as tablet and smartphone browsers.

The final version was approved by the professional associations (the Dutch Society of Obstetrics and Gynaecology and the Dutch Paediatric Society). The DA was also approved by the Dutch patient organization (CARE4NEO). Furthermore the DA was given the “Quality seal of comprehendible texts” by the Dutch Easy Reading Platform.

The DA (in Dutch) was made available on www.keuzehulpvroeggeboorte.nl in the autumn of 2019.

## Discussion and conclusion

### Discussion

This study describes the development of a DA to support prenatal counseling in extreme prematurity with involvement of healthcare professionals, parents who experienced extreme prematurity, and (potential) future users. Its content is in line with the Dutch guideline for perinatal management of extremely preterm delivery and the nationwide recommendations for counseling on this matter [12, 16]. We followed the developmental process according to the International Patient Decision Aid Standards [21]. Visual additions, like pictures and pictographs, were included and where applicable, visuals and numbers of mortality and morbidity were made optional, expanding in boxes upon clicking. Besides solely providing condition relevant information, our DA provides decision support as an additional tool. This differentiates our DA from existing DAs as described in Supplementary material Additional file 1. Thanks to the critical input of both clinicians as well as patient participants, in alpha and beta testing, the final version DA was well accepted and found easy to use, and it fulfilled all IPDAS quality criteria [24]. The format of the digital DA is an open access.

The following strengths of this study need to be discussed. This DA is developed with a systematic approach as recommended by IPDAS [21]. Parts of the development, as described by IPDAS, were performed in an extensive research program, published as the Dutch counseling recommendations [12]. This current study continued to involve both patients and clinicians in order to develop a DA that is well accepted by our target audience. By adding steps of decision support and value clarification, and by adding expandable, optional text and figures, this DA tries to facilitate personalization by responding to specific informational needs of expectant parents and by exploring personal values. The involvement of end-users enhanced representativeness and promotes future implementation, also underlined by approval of professional societies and patient organisation.

There are limitations to this study. Due to the national setting, it is unknown to which extent our content is exemplary for other, international settings. The use of Dutch outcome data can be regarded as cultural-sensitive, since the target population is Dutch, and Dutch health care providers wanted national data. However, others may be worried that these outcome data introduce a bias since the restrictive Dutch approach could influence outcome data [29, 30]. I.e. the restrictive approach may cause lesser babies of 23 and 24 weeks to survive, which in turn may influence decision-making in these categories when one believes that these babies ‘hardly survive’. Our development process, and general content could probably be used cross-cultural. Although we used experts for readability of the text, our beta testing was not specifically targeted to parents with lower education or limited reading skills (approximately 9% of Dutch population [Statistics Netherlands]). As of now, the DA is available in Dutch language only.

### Practice implications

In the counseling of parents facing imminent extreme premature labor, practice variation exists as a result of different health care systems and various cultural, social and individual preferences [31]. The decision between palliative comfort care or early intensive care is value-loaden, and a so called *preference sensitive* decision. Parents need to understand the options, the risks and benefits of each option, and the related information about potential outcomes. This information must be presented in a balanced way and physicians should elicit and clarify individual values of the parents facing this decision [10–12]. Decision-making may benefit from tools as our newly-developed digital DA. The use of our DA is not intended to replace the actual counseling conversation, but to support clinicians and parents in this sensitive situation [12, 17, 18]. Parents can make use of the DA if they wish to, before or even after the counseling conversation takes place.

In contrast, restraints have been described for the use of DAs in this setting. Although parents are usually involved in development and design of DAs, as in ours, it is unsure whether its information will match the information need of all parents [5, 32]. Furthermore, risk perception and the relative lack of data on quality of life and adjustment/resilience of families may color the information presented in Das [5, 29]. It is known that health care providers and parents can have different views regarding important information and outcomes [33–36]. From the clinician perspective, health care providers may have different attitudes towards shared decision making or DAs in general [27].

Finally, research on how to elicit patient values in this setting is scarce but growing, and should be incorporated in future updates of our DA [4, 10, 37].

The overview of existing DAs, as shown in Additional file 1, showed the different types of tools that are available worldwide on this subject. In most studies, both patients as well as clinicians were involved in the development. Some described DA’s focus (more) on dissemination of medical information rather than helping parents’ to explore their values and preferences [4]. Evaluations showed increased knowledge of extreme premature delivery and its sequelae. Furthermore, most published DAs on extreme premature delivery are card illustrations for additional information during the consultations [15, 18, 19, 21]. Focusing on the format and distribution of our DA, we developed a freely available online DA and, moreover, included features to support decision making and help parents think about their values and preferences. This is anticipated to enhance the uptake and use of the DA amongst different types of users, and we hope to increase the involvement of parents in decision-making.

The effectiveness of DAs in decision-making has already been proven on many different levels [13, 14]. Future studies should focus on increasing the uptake of DAs by those who wish to use them. Identifying the barriers and facilitators to implementation is also important in increasing their relevance and application. Also, further understanding is needed of which values parents have, how they develop and how they are best elicited—it is of utmost importance to adjust and update supportive tools according to this knowledge [4, 10, 38].

## Conclusion

A digital DA to support prenatal counseling regarding the decision on early intensive care versus palliative comfort care at imminent extreme premature delivery was developed and was well accepted by parents and health care professionals during beta testing. By following a systematic developmental process with critical involvement of both clinicians and (future) parents, the digital DA is anticipated to support the decision-making process. Implementation of this digital DA, as well as its effects on shared decision making, is the focus for future studies.

## Supplementary Information

**Additional file 1**. Overview of existing DA tools for imminent extremely premature delivery.

**Additional file 2**. Supplemental DA figures.

**Additional file 3**. International Patient Decision Aid Standards instrument.

## Data Availability

Data sharing is not applicable to this article as no datasets were generated or analysed during the current study.

## Abbreviations

DA: Decision aid
GA: Gestational age
IPDAS: International patient decision aids standards
NICU: Neonatal intensive care unit
SDM: Shared decision making
