# Supplementary material for: Digital decision aid for prenatal counseling in imminent extreme premature labor: development and pilot testing
Source: BMC Med Inform Decis Mak. 2022 Jan 6;22:7. doi: 10.1186/s12911-021-01735-z (PMC8734286; doi:10.1186/s12911-021-01735-z)
Supplement: Supplementary file 1 — Additional file 1. Overview of existing DA tools for imminent extremely premature delivery. [file 12911_2021_1735_MOESM1_ESM.docx]

**Additional file 1. Overview of existing DA tools for imminent extremely premature delivery**

|  | **Description of the tool** | **Involved in design of the tool** | **Evaluation of the tool** |
| --- | --- | --- | --- |
| **Kakkilaya et al, 2011**  **(USA)** | Visual aid with relevant graphics and short, easy-to-read messages | Not described | 89 women with GA >28, randomized between counseling in a hypothetical scenario of delivering at 23 weeks, with and without visual aid.  Significant improvement of knowledge in the DA group. |
| **Guillen et al, 2012**  **(USA)** | Card set with pictures & pictographs showing survival rates and complications of intensive care | 31 clinicians, 30 parents of children born <26 weeks GA | 13 “experienced” parents and 11 “naive” women.  Significant improvement of knowledge |
| **Guillen et al, 2016**  **(USA)** | 10 minute video with clips of NICU admission, toddlers with several impairments and parents explaining their decision | See Guillen et al (2012) | 16 clinicians, 14 “experienced” parents and 13 “naive” women. Video was well accepted without increase of anxiety. |
| **Moore et al, 2017**  **(Canada)** | Modified card aid of Guillen et al (2012), updated following IPDAS criteria. A palliative care card, and cards on quality of life and maternal impact were added. | See Guillen et al (2012), along with 17 multi-stakeholders including parents | 20 participants at risk of delivering between 23 and 24 weeks GA; 89% of them recommending this form of consultation.  Decisional conflict decreased. |
| **Drago et al, 2018**  **(USA)** | Spanish language DA cards based on the cards of Guillen et al (2012) | See Guillen et al (2012), along with 22 Latino parents with history of birth <26 weeks GA | 9 “experienced” parents and 10 “naive” volunteers.  DA was well received and increased knowledge in “naïve” parents. |
| **Tucker Edmonds et al 2018**  **(USA)** | Three prototypes: a tablet application, family story videos and a virtual reality experience | 48 mothers/partners experienced with birth <25 weeks GA, and 17 physicians and 30 (obstetric/neonatology) nurses | 17 representatives evaluated three prototypes.  The app was found most feasible, effective and desirable. |
| **Guillen et al, 2019**  **(USA)** | The 6-card DA of Guillen et al (2012) | See Guillen et al (2012) | Randomized trial in 201 women with imminent premature delivery (GA 22-26 weeks). Decisional conflict scores were similar but knowledge improved significantly after counseling with the DA. |

(DA decisional aid; GA gestational age; IPDASi International Patient Decision Aid Standards instrument; USA United States of America).
