# Supplementary material for: Digital decision aid for prenatal counseling in imminent extreme premature labor: development and pilot testing
Source: BMC Med Inform Decis Mak. 2022 Jan 6;22:7. doi: 10.1186/s12911-021-01735-z (PMC8734286; doi:10.1186/s12911-021-01735-z)

**Additional file 2. Supplemental DA figures**

Figure 1. Landing page of the digital DA, in Dutch. The title of this page is “What is imminent extreme preterm labor”

The button with “Informatie” in pink (English: Information) contains Step 1 to 6 of the DA (See Table 2 in the manuscript). Next to this button are the buttons of Step 7 (Comparison Page), Step 8 (Important points) and Step 9 (My choice).


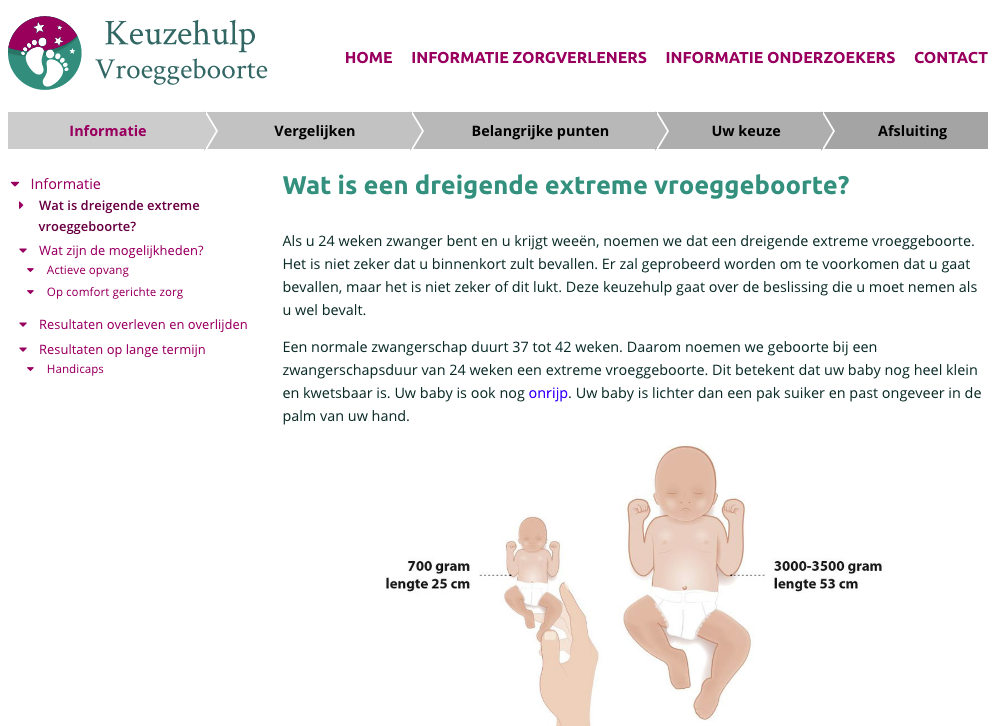


Figure 2. Step 9 of the DA: the “My choice” page. This Step includes a values clarification exercise with 7 propositions to weigh the two different treatment options in line with the users’ values. Each time, two contrasting propositions are presented, and the user can swipe to the left (early intensive care) or right (palliative comfort care) to show her/his feeling about this subject.


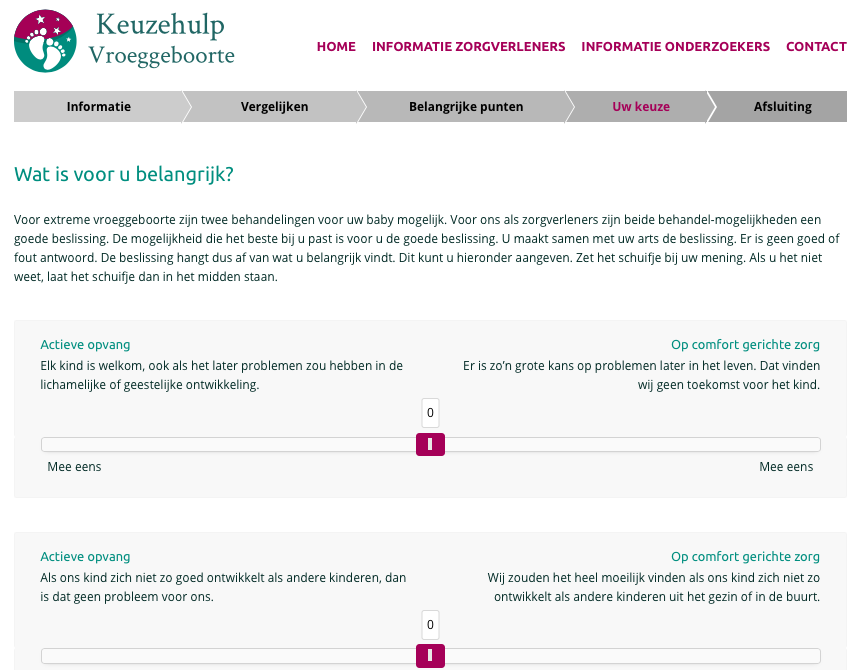

Supplement: Supplementary file 2 — Additional file 2. Supplemental DA figures. [file 12911_2021_1735_MOESM2_ESM.docx]
