# Supplementary material for: Digital decision aid for prenatal counseling in imminent extreme premature labor: development and pilot testing
Source: BMC Med Inform Decis Mak. 2022 Jan 6;22:7. doi: 10.1186/s12911-021-01735-z (PMC8734286; doi:10.1186/s12911-021-01735-z)
Supplement: Supplementary file 3 — Additional file 3. International Patient Decision Aid Standards instrument. [file 12911_2021_1735_MOESM3_ESM.docx]

**Additional file 3. International Patient Decision Aid Standards instrument**

Reference:

Joseph-Williams N, Newcombe R, Politi M, Durand MA, Sivell S, Stacey D, et al. Toward Minimum Standards for Certifying Patient Decision Aids: A Modified Delphi Consensus Process. Med Decis Making. 2014;34(6):699-710.

| **Type of criterium**  Qualifying criteria (QfC)  -  Certfication Criteria (CC)  -  Quality Criteria (QtC) | **Criterium** | **Location**  **(See Table 2 of manuscript)** |
| --- | --- | --- |
| **Information (Providing information about options in sufficient detail for making a specific decision )** | | |
| QfC | The patient decision aid describes the health condition or problem (intervention, procedure or investigation) for which the index decision is required | DA (Step 1) |
| QfC | The patient decision aid explicitly states the decision that needs to be considered (the index decision) | DA (Step 2) |
| QfC | The patient decision aid describes the options available for the index decision | DA (Step 2) |
| QfC | The patient decision aid describes the positive features (benefits or advantages) of each option | DA (Step 3-6) |
| QfC | The decision aid describes negative features (harms, side effects or disadvantages) of each option. | DA (Step 3-6) |
| CC | The patient decision aid shows the negative and positive features of options with equal detail (e.g. using similar fonts, order, and presentation of statistical information). | DA (Step 3-7) |
| QtC | The patient decision aid describes the natural course of the health condition or problem, if no action is taken (when appropriate). | DA (Step 4) |
| QtC | The patient decision aid makes it possible to compare the positive and negative features of the available options. | DA (Step 7) |
|  |  |  |
| **Probabilities (Presenting outcome probabilities)** | | |
| QtC | The patient decision aid provides information about outcome probabilities associated with the options (i.e. the likely consequences of decisions) | DA (Step 3-6) |
| QtC | The patient decision aid specifies the defined group (reference class) of patients for which the outcome probabilities apply. | DA (Step 3-6) |
| QtC | The patient decision aid specifies the event rates for the outcome probabilities. | DA (Step 3-6) |
| QtC | The patient decision aid allows the user to compare outcome probabilities across options using the same time period (when feasible). | DA (Step 3-6) |
| QtC | The patient decision aid allows the user to compare outcome probabilities across options using the same denominator (when feasible). | DA (Step 3-6) |
| QtC | The patient decision aid provides more than one way of viewing the probabilities (e.g. words, numbers, and diagrams). | DA (Step 3-7) |
|  |  |  |
| **Values (Clarifying and expressing values)** | | |
| QfC | The patient decision aid describes what it is like to experience the consequences of the options (e.g. physical, psychological, social) | DA (Step 3-6, 9) |
| CtC | The patient decision aid asks patients to think about which positive and negative features of the options matter most to them (implicitly or explicitly). | DA (Step 3-6, 9) |
|  |  |  |
| **Decision Guidance (Structured guidance in deliberation and communication)** | | |
| QtC | The patient decision aid provides a step-by-step way to make a decision. | DA (Step 1-9) |
| QtC | The patient decision aid includes tools like worksheets or lists of questions to use when discussing options with a practitioner. | DA (Step 7-9) |
|  |  |  |
| **Development (Using a systematic development process)** | | |
| QtC | The development process includes a needs assessment with clients or patients | yes, see Manuscript |
| QtC | The development process included a needs assessment with health professionals | yes, see Manuscript |
| QtC | The development process included expert review by clients/patients not involved in producing the decision support intervention | yes, see Manuscript |
| QtC | The development process included expert review by health professionals not involved in producing the decision support intervention | yes, see Manuscript |
| QtC | The patient decision aid was field tested with patients who were facing the decision. | yes, see Manuscript |
| QtC | The patient decision aid was field tested with practitioners who counsel patients who face the decision. | yes, see Manuscript |
|  |  |  |
| **Evidence (Using evidence)** | | |
| CC | The patient decision aid (or associated documentation) provides citations to the evidence selected. | yes, see Manuscript |
| CC | The patient decision aid (or associated documentation) provides a production or publication date. | DA (Introduction) |
| CC | The patient decision aid (or associated documentation) provides information about the update policy. | DA (Step 5), Manuscript |
| CC | The patient decision aid provides information about the levels of uncertainty around event or outcome probabilities (e.g. by giving a range or by using phrases such as ‘‘our best estimate is…’’) | DA (Step 5) |
| QtC | The patient decision aid (or associated documentation) describes how research evidence was selected or synthesized. | DA (Step 5), Manuscript |
| QtC | The patient decision aid (or associated documentation) describes the quality of the research evidence used. | Manuscript and references |
|  |  |  |
| **Disclosure** | | |
| CC | The patient decision aid (or associated documentation) provides information about the funding source used for development. | DA (Introduction) |
| QtC | The patient decision aid includes author/developer credentials or qualifications. | DA (Introduction) |
|  |  |  |
| **Plain Language** | | |
| QtC | The patient decision aid (or associated documentation) reports readability levels (using one or more of the available scales). | DA (Introduction) |
|  |  |  |
| **DST Evaluation** | | |
|  | There is evidence that the patient decision aid improves the match between the features that matter most to the informed patient and the option that is chosen | Manuscript |
|  | There is evidence that the patient patient decision aid helps patients improve their knowledge about options’ features | Manuscript |
| **Test** *(not applicable)* | | |
